# Rotational Atherectomy-Assisted Crossing of a Complex Right Coronary Artery Lesion Using the Retrograde Approach

**DOI:** 10.1155/2021/8832213

**Published:** 2021-08-26

**Authors:** Abdul-Subulr Yakubu, Xiaoqiang Zhang, Bin Zhang

**Affiliations:** ^1^Tamale Teaching Hospital, Tamale, Ghana; ^2^Chengdu 2nd People Hospital, China; ^3^Second Division of the Cardiology Department, Guangdong Cardiovascular Institute, Guangdong General Hospital, Guangdong, China

## Abstract

Chronic total occlusion lesions present a major challenge for the interventional cardiologist. In this case, we report the successful use of rotational atherectomy to facilitate retrograde percutaneous coronary intervention of a complex totally occluded right coronary artery after modification of the proximal cap of the lesion to enable placement of the RotaWire in the vessel architecture.

## 1. Introduction

The results of percutaneous coronary interventions (PCI) have dramatically improved in the last decades. Despite this improvement, chronic total occlusion (CTO) lesions still remain a major challenge of interventional cardiology due to the complexity of these lesions. As CTO equipment and techniques continue to improve, so has the success of wiring occlusive lesions [1]. Rotational atherectomy (RA) in CTO has found use in situations where balloon or microcatheter advancement poses a challenge following successful guide wire crossing. This approach has been found to be feasible and safe and to increase the procedural success rate in these tough CTO lesions with heavy calcification [2]. However, its role in heavily calcified wire-uncrossable lesions is less well established and RA is generally considered contraindicated in these situations.

## 2. Case Presentation

**Figure 1:**
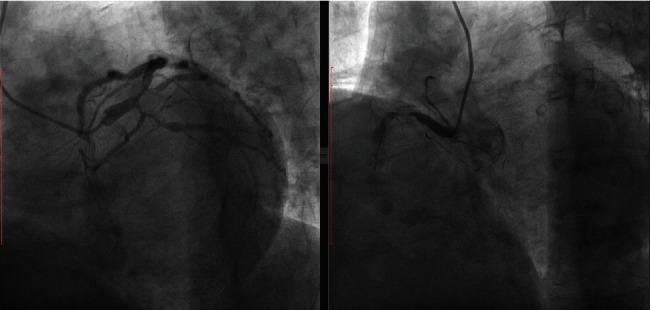
(a) Coronary angiogram of the LCA showing a severe bifurcation lesion involving the proximal LAD and LCX. (b) RCA angiogram shows a proximal CTO with a diffusely calcified vessel. There is a broken guide wire tip in the mid portion of the RCA from the previously failed PCI attempt.

The patient is a 68-year-old male with chronic kidney disease on peritoneal dialysis for 3 years. He presented with recurrent exertional chest pain at a local facility where he underwent coronary angiography showing complex 3-vessel disease with severe diffuse calcification of all coronary arteries. The proximal left anterior descending (LAD) and left circumflex (LCX) arteries showed 99% stenosis, and the right coronary artery (RCA) was totally occluded (Figure 1). A broken coronary guide wire was left in situ following a failed attempt at RCA revascularization.

**Figure 2:**
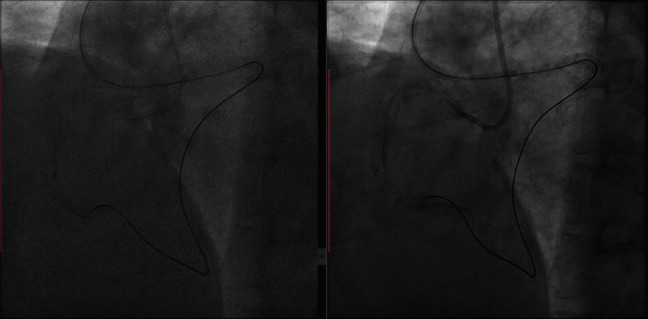
(a) Rotational atherectomy through the vessel architecture of the proximal segment of the RCA CTO lesion. (b) Guide extension catheter (Guidezilla) placed through antegrade guiding catheter helps with tracking the retrograde guide wire into the antegrade guiding catheter.

The patient was referred to the Guangdong Cardiovascular Institute for angioplasty. The RCA was engaged with a 7-French Amplatz left 0.75 guiding catheter and the left coronary artery (LCA) with 7-French XB 3.5 catheter. Antegrade crossing of the RCA chronic total occlusion (CTO) lesion with various guide wires including Pilot 150 and 200, UB3, and Conquest Pro failed necessitating a switchover to the retrograde strategy. The LAD lesion was prepared with rotational atherectomy, and the proximal segment was stented with a 3.0∗24 mm drug-eluting stent (DES). The RCA was approached retrogradely through the first septal collateral using SION blue guide wire through a 150 cm Corsair microcatheter (Asahi Intecc, Nagoya, Japan) and advanced to the distal cap of the CTO lesion. A Pilot 150, Pilot 200, UB3, and Conquest Pro guide wires failed to cross to the proximal true lumen. Through the antegrade guide and with the help of stiffer guide wires (Pilot 200, Conquest Pro), the proximal cap of the CTO lesion was punctured and modified. This eventually facilitated the placement of a RotaWire (Boston Scientific, Marlborough, MA, USA) through the architecture of the occluded vessel followed by rotational atherectomy with a small-sized (1.25 mm) burr. Repeated cycles of proximal cap modification with further advancement of the RotaWire and rotational atherectomy through the vessel architecture facilitated passage of the antegrade gear (microcatheter and guide extension catheter) through the long CTO segment (Figure 2).

**Figure 3:**
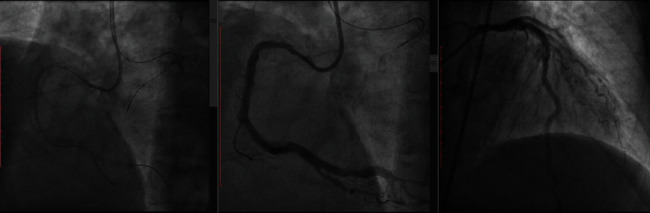
(a) Successful wiring of RCA into PD and PL blanches. PD = posterior descending; PL = posterolateral. (b) RCA angiogram postprocedure. (c) Left angiogram postprocedure.

Reverse controlled antegrade and retrograde subintimal tracking (reverse CART) facilitated passage of the retrograde guide wire into the antegrade guide extension (Guidezilla, Boston Scientific) and guiding catheter. A successful “rendezvous” between the retrograde microcatheter and an antegrade RotaWire (Boston Scientific, Marlborough, MA) was achieved, followed by further rotational atherectomy into the distal true lumen. The procedure was completed using standard techniques after exchanging for a workhorse guide wire. Three DES were implanted into the RCA with satisfactory final results (Figure 3). The whole procedure lasted 03:04 hours of which 02:11 hours were spent on the RCA lesion. About 260 milliliters of contrast agent was used.

## 3. Discussion and Conclusion

Flexibility, creativity, and patience are often required to achieve success in CTO PCI as each CTO lesion is unique. Retrograde CTO PCI has revolutionized CTO percutaneous coronary intervention and in experienced operators is as effective and safe as an antegrade approach [3–5].

Rotational atherectomy has found use in balloon or microcatheter uncrossable lesions, which are a relatively common occurrence in CTO PCI. Whilst the feasibility of RA in CTO PCI has already been suggested, current practice and recommendations dictate that an attempt at RA should follow confirmation of placement of the guide wire in the true lumen [6]. Rotational atherectomy through a dissection plane is generally contraindicated because of concerns of expansion of the dissection plane and vessel perforation. Recently, however, it has been demonstrated that RA can be used successfully provided one remains in the vessel architecture and employs a small- to medium-sized burr [7, 8]. Rotational atherectomy in CTO has been found to be safe and effective with outcomes not adversely affected by the presence of a prior dissection [9].

Not infrequently, particularly in long, tortuous, and calcified lesions, as in our case, a true-to-true lumen wiring is unsuccessful. The concept of “vessel architecture” is based on the distinction between coronary structures (plaque, intima, media, and adventitia) and the extravascular space and represents a safe work environment for guide wire and device manipulation [10]. Hence, the ability to manage the subadventitial space is invaluable in contemporary CTO PCI. In the absence of intravascular imaging, the location of the guide wire within the vessel architecture in our case was guided by the tactile feedback of the wire as well as the contralateral angiogram. Operator experience in such situations is key to producing a successful outcome as RA in the adventitia (versus subintimal or intraplague) is associated with a higher likelihood of perforation. The operator in our case is a very experienced interventionist who has performed over one thousand retrograde PCI procedures.

Our case demonstrates the successful use of RA through a calcified and totally occluded RCA segment to facilitate retrograde PCI after an initial failure of the traditional antegrade and retrograde techniques. The proximal cap of the CTO lesion was modified with high tip-load guide wires to facilitate placement of the RotaWire (Boston Scientific) within the vessel architecture of CTO segment followed by successful RA and retrograde crossing by reverse CART.

## Data Availability

The data used to support the findings of this study are available from the corresponding author upon request.
